# Reducing Health Inequities in Australia: The Role of Place‐Based Action and Spatial Data Infrastructure

**DOI:** 10.1002/hpja.70156

**Published:** 2026-01-21

**Authors:** Jonathan R. Olsen, Mark Robinson, Jonathan Corcoran, Lisa McDaid

**Affiliations:** ^1^ Institute for Social Science Research The University of Queensland Brisbane Australia; ^2^ School of the Environment The University of Queensland Brisbane Australia

**Keywords:** Australia, health inequities, life expectancy, median age of death, place‐based health, spatial inequities

## Abstract

Australia holds one of the world's highest life expectancies, yet stark geographic inequities persist. In Queensland, the gap in median age of death between communities only 85 km apart reaches 26 years for men and 22 years for women. While national policies increasingly endorse “health‐in‐all‐policies” and place‐based approaches, their impact depends on the ability to identify, target and monitor inequities at fine geographic scales. Spatially referenced data and analytic techniques provide a critical foundation for this work, enabling baseline assessments, tailored interventions, and rigorous evaluation. To fully realise this potential, commonwealth and federal governments must commit to sharing spatially open data, invest in capacity‐building for its use, and foster transdisciplinary collaboration. Without these supports, the promise of place‐based health perspectives to tackle health inequities will remain unrealised.

## 1 Introduction

Australia ranks among the countries with the highest life expectancy globally, placing fourth out of the 38 member nations of the Organisation for Economic Co‐operation and Development (OECD) [1]. Nationally, the average life expectancy is 85 years for females and 81 years for males [2]. However, significant geographic inequities in life expectancy persist across Australian states and territories. For males, life expectancy is highest in the Australian Capital Territory (81.7 years) and lowest in the Northern Territory (76.4 years). Among females, it is highest in Western Australia (85.7 years) and lowest in the Northern Territory (80.4 years) [3]—a gap of up to 5 years between jurisdictions.

There is an 8.8‐year difference in life expectancy between Indigenous (71.9 years males; 75.6 years females) and non‐Indigenous (80.6 years males; 83.8 years females) populations across Australia [4]. Colonialization and a range of deep‐rooted systemic factors have contributed to these inequities [5]. This gap is especially pronounced in remote areas where the proportion of the population who are First Nations is 47% compared to 2.2% within major cities [6], compounding the geographic inequities examined in this commentary.

Australia has a particularly distinctive geography. As the sixth largest country in the world by land area (7 688 287 km^2^), it has a relatively small population of 27.2 million [7]. With an average population density of only 3.5 people per km^2^ [8], the majority (68%) live in the eight capital city regions, which occupy less than 1% of the total landmass.

This uneven population distribution—marked by sparsely populated remote areas and highly urbanised capital cities—poses complex challenges for addressing premature mortality and achieving equitable health outcomes.

To help us better understand the complex challenge of achieving place‐based health equity, this commentary examines within‐city and state‐wide inequities in median age of death in Queensland, Australia—a state of 1 729 742 km^2^, encompassing the full range of urbanised, regional and remote areas seen across Australia [7]. It broadly explores policy relevant place‐based approaches for improving population health and tackling geographic health inequities, provides examples of how spatial and urban data infrastructure can contribute to the effective implementation and evaluation of place‐based health interventions, and finally highlights further opportunities and challenges of these approaches. The core contribution of this commentary is to highlight how spatial data infrastructure can identify geographic inequalities, support place‐based action, monitoring and evaluation, as well as future opportunities and challenges.

## 2 Spatial Variation in Median Age of Death in Queensland: State‐Wide and Within Cities

Life expectancy—and median age of death, as shown here—are key indicators of population health and wellbeing. These metrics reflect the social, economic, and environmental conditions in which people live and can reveal stark inequities in health outcomes across different geographic areas [9].

**FIGURE 1:**
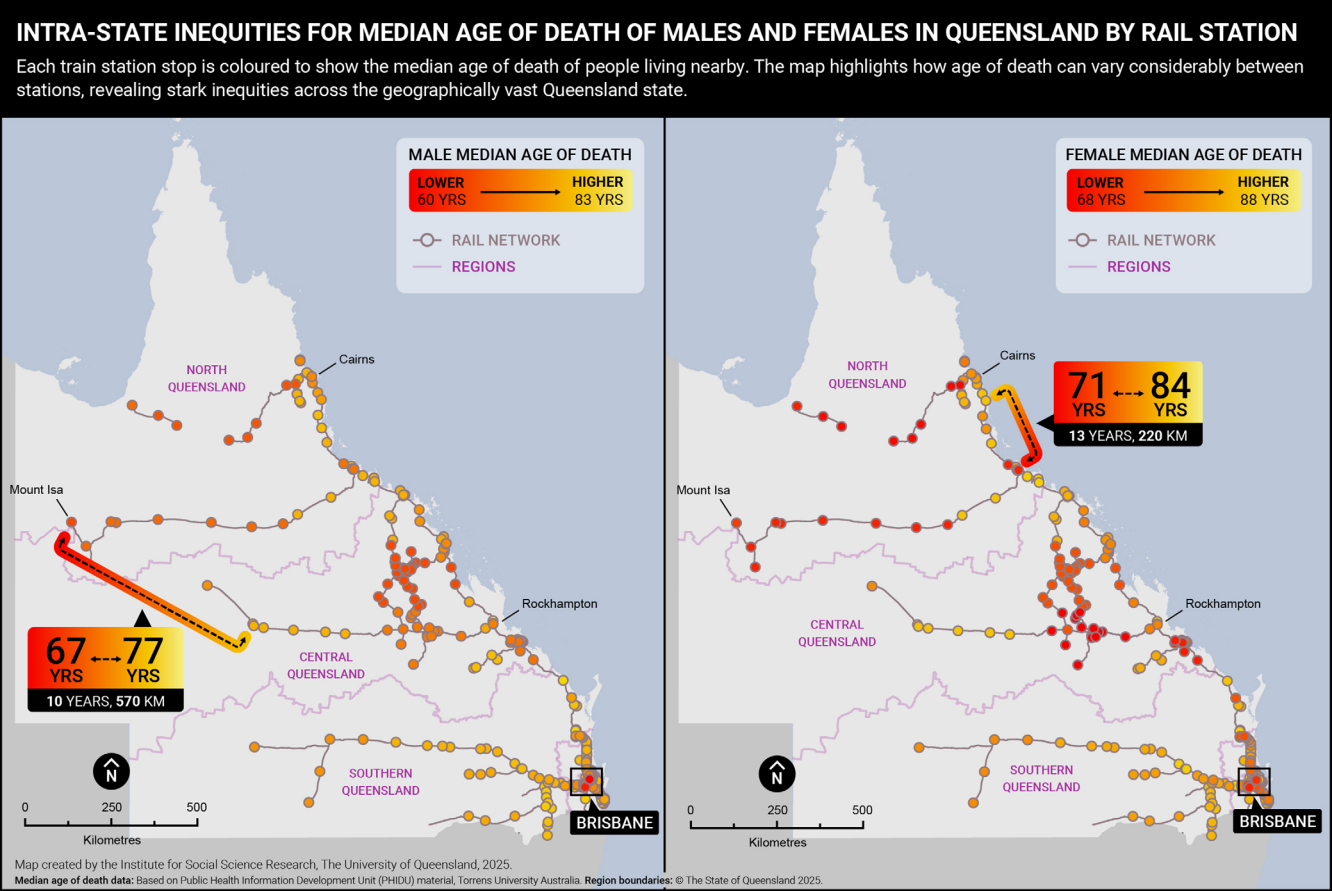
Male and female median age of death (2018–2022) by Train Station location, Queensland. Based on Public Health Information Development Unit (PHIDU), Torrens University Australia material from: Social Health Atlas of Australia: Queensland (online) 2025. Accessed date 12/07/2025. Each point represents a Statistical Area 2 (SA2) containing a train station.

Figure 1 presents the median age of death for males and females across Queensland, mapped in relation to the state's rail network—a technique previously used to visualise spatial inequities in population health in Glasgow, Scotland [10]. Visualising inequities via maps or infographics is a key tool to provoke critical thinking and debate about the causes, consequences, and strategies for reducing inequities [11]. The maps show that residents in South East Queensland, encompassing Brisbane, the Gold Coast, and the Sunshine Coast, generally experience higher life expectancy than those living in central and northern regions of the state. For example, males living in Springfield Lakes have a median age of death of 59 years, 26 years lower than their counterparts in Clear Island Waters (85 years). Among females, the difference between these same locations is 22 years (67 vs. 89 years) [12]. These locations are separated by only 85 km—highlighting how large health inequities can exist across relatively short distances in a geographically vast state.

**FIGURE 2:**
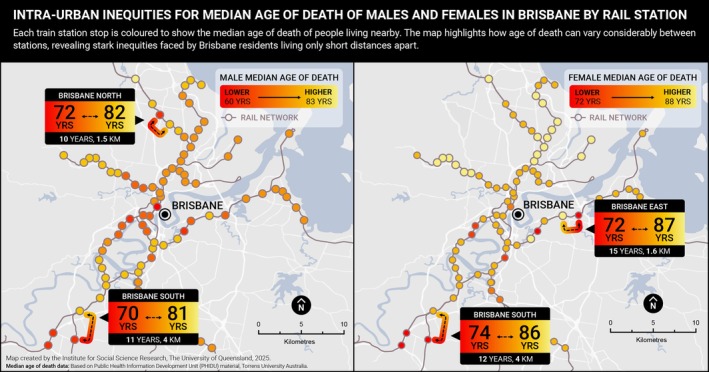
Male and female median age of death (2018–2022) by Train Station location, Brisbane City. Based on Public Health Information Development Unit (PHIDU), Torrens University Australia material from: Social Health Atlas of Australia: Queensland (online) 2025. Accessed date 12/07/2025. Each point represents a Statistical Area 2 (SA2) containing a train station.

Marked inequities are also evident within cities. In Brisbane, for example, the median age of death for males ranges from 60 to 84 years—a 24‐year gap. For females, the difference is 17 years (72 vs. 89 years) [12]. Figure 2 illustrates these intra‐urban inequities, using train stations as geographic markers to demonstrate proximity. Communities located just five train stops apart—less than 5 km by road—can have over 20 years' difference in median age of death for males.

## 3 Health Inequities in Australia

In Australia, the National Preventive Health Strategy aims to give communities the best start in life, support individuals to live longer and healthier lives, address health inequities between socio‐economic groups, and prioritise prevention over treatment [13]. Central to this strategy is the recognition that health is shaped by a broad range of contextual factors—including social, environmental, economic, cultural, and commercial conditions—collectively known as the *wider determinants of health*. The strategy further embeds this understanding through its policy commitment to a Health in All Policies (HiAP) approach by 2030. This whole‐of‐government strategy acknowledges the interconnectedness of policy domains and promotes coordinated action across all levels of government to reduce health inequities and improve population health [14].

## 4 Place‐Based Approaches to Tackle Health Inequities

Place‐based approaches—particularly those that are co‐designed with communities and adopt community‐grounded models—are proposed as strategies for improving health outcomes in disadvantaged areas and reducing health inequities [15]. These approaches focus on specific geographic areas that are ‘underperforming’ in terms of health outcomes [16], and involve collaboration between local stakeholders, service providers, and residents to address the complex social and environmental determinants of health [17]. They acknowledge that health outcomes vary significantly depending on where people live and are gaining increasing attention and adoption by governments worldwide [18], including Australia. These approaches align to the social determinants of health, which highlight that health outcomes are influenced by a broad range of individual, social, community, environmental, and political factors [19]. By involving communities directly, place‐based initiatives aim to empower individuals, leverage local knowledge, and foster community‐led solutions that are tailored, acceptable, and sustainable. In addition to improving physical health and health behaviours, these initiatives can strengthen social cohesion, reduce isolation, and enhance collective self‐efficacy [20]. These incentives may be particularly relevant for rural and remote areas, which have been shown to receive less healthcare funding per person compared to urban residents [21].

Examples of place‐based initiatives include enhancing local amenities, expanding active travel infrastructure, and improving the quality of public open spaces. These efforts have demonstrated positive impacts on physical activity, mental wellbeing, and broader social determinants of health [17].

Australia has seen promising examples of such initiatives. The *Tackling Indigenous Smoking* programme, for instance, invested in 37 regional teams to deliver culturally appropriate, community‐led health promotion activities. Since its implementation, the programme has contributed to a notable reduction in smoking prevalence, particularly among younger Aboriginal and Torres Strait Islander people, which has reduced smoking inequities between non‐Indigenous populations [22].

In 2023, the Australian Government announced a $200 million funding initiative to address *entrenched intergenerational disadvantage and improve child and family wellbeing* [23]. At the core of this initiative is the use of place‐based partnerships, with an emphasis on co‐designing solutions that respond to the specific needs and priorities of local communities.

To evidence the impact of such initiatives, monitoring and evaluation are essential for learning what works, what doesn't, and why. However, global reviews of place‐based interventions have identified a recurring challenge: insufficient data infrastructure for monitoring and evaluation [17]. Without reliable data and appropriate outcome measures, it remains difficult to identify priority areas, assess the long‐term impacts of initiatives on health inequities, and to build a robust evidence base for scaling up successful models.

## 5 Using Geospatial and Urban Data Infrastructure to Support Monitoring, Evaluation, and Place‐Based Action on Health Inequities

Geographical and urban data infrastructure can play a critical role in supporting the design, monitoring, and evaluation of place‐based approaches aimed at improving population health and reducing health inequities. Spatially referenced data—particularly when disaggregated to small geographic areas—can uncover inequities, track change over time, and guide targeted action. These tools also support the development of evidence‐informed policies by linking health outcomes with environmental, demographic, and infrastructure data.

Table 1 outlines the key domains in which spatial and urban data infrastructure can contribute to the effective implementation and evaluation of place‐based health initiatives. It demonstrates the range of ways in which such data infrastructure could help evidence the impact of Australian Government initiatives at national, state/territory and local levels. These data sources and tools can also be useful to evaluate and monitor key aspects of the social determinants of health for small areas, such as demographic, health care access and availability, environmental, employment, social and political/governance characteristics. However, there do remain current limitations in the integration of such data infrastructure in Australia.

**TABLE 1:** Domains, descriptions and examples of how spatial and urban data infrastructure can contribute to the effective implementation and evaluation of place‐based health interventions.

Domain	Description	Example
Place‐based health and social needs assessments	Spatially referenced health data, visualised using Geographic Information Systems (GIS), can highlight stark intra‐urban and regional inequities. Local‐level data, such as median age of death, help identify priority areas for intervention.	*Health service planning*: GIS was used to map areas with low access to primary care services, based on population health needs and sociodemographic characteristics [24].
Integration of small‐area social, health, and environmental data	Combining ABS census data with local health and environmental datasets enables monitoring of population changes, local context, and health outcomes over time.	*Infrastructure evaluation*: Travel diary data collected pre‐ and post‐opening of new road infrastructure were analysed to compare active travel behaviours across intervention, comparator, and control areas [25].
Environmental monitoring	Spatial data can track environmental changes—such as greenspace provision, housing conditions, and transport infrastructure—that may influence population health.	*Built environment change*: National mapping agency data were used to categorise small areas as having no change, loss or gain, in buildings, roads, and woodland over time, and their impact on health outcomes [26].
Urban planning and land use assessment	Land use and urban infrastructure data can be used to examine how features of the built and natural environment influence health and health‐related behaviours.	*Ecological assessment*: Urban tree canopy coverage was analysed alongside area‐level health and demographic data to explore associations with mental health and wellbeing [27].
Policy modelling	Spatial modelling can simulate the potential impact of future urban policies, particularly those targeting health‐related environments.	*Advertising policy modelling*: GIS was used to assess the potential impact of restricting unhealthy commodity advertising within proximity to schools, comparing current exposure with projected outcomes under proposed policy changes [28].
Data dashboards and visualisation tools	Interactive dashboards built on GIS platforms can visualise health, social, and environmental data for small areas. These tools support policymakers, communities, and advocacy groups in understanding local conditions and driving place‐based action.	*Census mapping*: National health dashboards displaying population, education, housing and health data at census output areas [29].

## 6 Opportunities and Challenges in Integrating Geospatial Data to Address Health Inequities

The integration of geographical data into health equity and social science research, as well as policy, presents significant opportunities but also faces ongoing challenges that must be addressed to unlock its full potential.

Australia's growing geospatial data infrastructure provides a strong foundation for supporting place‐based approaches to health. The Australian Urban Research Infrastructure Network (AURIN) (https://aurin.org.au/) plays a central role in this ecosystem by streamlining access to diverse datasets—including health, transport, housing, economic, demographic, and land use data—for use by researchers, government, and industry. AURIN serves as a national knowledge broker, offering a single point of access to spatial data and facilitating evidence‐informed decision‐making.

Other initiatives include the Academy of Social Sciences Decadal Plan for Social Science Research Infrastructure, and the recently funded Social Science Research Infrastructure Network (SSRIN), which will bring together academics, government analysts, data custodians, and policy makers to access the data and tools needed to understand and address the pressing and complex needs of our society.

Despite these advancements, several barriers persist. A key limitation is the restricted availability of open‐source spatial data at fine geographic and temporal resolutions. To support robust analyses of health inequities, national and state‐level government agencies should prioritise the release of timely, disaggregated spatial data—including by key sociodemographic subgroups—linked to other health and social data to enable monitoring of trends and subgroup comparisons over time. The drivers of spatial inequities are complex and include a number of structural and historical factors that are both difficult to quantify and extend beyond administrative boundaries. Researchers should also be cautious against area stigmatisation through the identification or ranking of areas based on their health or social outcomes.

Conducting small‐area studies also requires specialised skills in spatial analysis, statistical interpretation, and public health translation. Without this expertise, there is a risk of misinterpretation or underutilisation of complex spatial datasets. This is particularly relevant in rural and remote areas with low population densities where administrative units cover vast geographical areas to ensure non‐disclosure of individuals, which can subsequently lead to spatial exposure misclassification.

## 7 Conclusions and Recommendations

Geographic and spatially referenced data play a critical role in identifying, monitoring, and evaluating health programmes and interventions, particularly in addressing health inequities. In this commentary, we have demonstrated how spatially referenced life expectancy data can be a powerful tool for revealing disparities in health across Queensland and within the city of Brisbane. Where national, open source and high quality social and health spatially referenced data, such as the PHIDU data used here, are available this provides an opportunity for the exploration of spatial inequalities in other Australian States and Territories.

Fully harnessing the potential of geographic perspectives in health research and policy requires:

- Open access to spatially disaggregated data from government sources to enhance transparency, equity, and usability for researchers, policymakers, and communities
- Investment in capacity‐building for researchers and practitioners in spatial epidemiology and urban health analytics, ensuring the workforce is equipped to analyse and apply spatial data effectively.
- Fostering multidisciplinary collaboration, integrating expertise from urban planning, data science, public health, and community organisations in the design and implementation of infrastructure and place‐based health initiatives.
- Support for long‐term partnerships between data infrastructure platforms such as AURIN and policymakers to ensure spatial data is consistently leveraged in the development of evidence‐based strategies aimed at reducing entrenched health disparities.

While there are clear opportunities for the use of geographic data, addressing associated challenges—such as data access, technical capacity, and cross‐sector coordination—will be essential. With sustained commitment, spatial data can become an integral asset in building healthier, more equitable communities.

## Funding

The authors have nothing to report.

## Conflicts of Interest

The authors declare no conflicts of interest.

## Data Availability

Based on Public Health Information Development Unit (PHIDU), Torrens University Australia material from Social Health Atlas of Australia: Queensland (online) 2025. Accessed date 12/07/2025.
